# Integrated transcriptome and metabonomic analysis of key metabolic pathways in response to cadmium stress in novel buckwheat and cultivated species

**DOI:** 10.3389/fpls.2023.1142814

**Published:** 2023-03-17

**Authors:** Dongao Huo, Ying Hao, Juan Zou, Lixia Qin, Chuangyun Wang, Dengxiang Du

**Affiliations:** ^1^ Guizhou Normal University, Guiyang, China; ^2^ College of Biological Sciences and Technology, Taiyuan Normal University, Taiyuan, China; ^3^ School of Life Science and Technology, Wuhan Polytechnic University, Wuhan, China; ^4^ College of Agriculture, Shanxi Agricultural University, Taiyuan, China

**Keywords:** buckwheat, cadmium (Cd) stress, metabolome, transcriptome, reactive oxygen species (ROS)

## Abstract

**Introduction:**

Buckwheat (*Fagopyrum tataricum*), an important food crop, also has medicinal uses. It is widely planted in Southwest China, overlapping with planting areas remarkably polluted by cadmium (Cd). Therefore, it is of great significance to study the response mechanism of buckwheat under Cd stress and further develop varieties with excellent Cd tolerance.

**Methods:**

In this study, two critical periods of Cd stress treatment (days 7 and 14 after Cd treatment) of cultivated buckwheat (Pinku-1, named K33) and perennial species (*F. tatari-cymosum* Q.F. Chen) (duoku, named DK19) were analyzed using transcriptome and metabolomics.

**Results:**

The results showed that Cd stress led to changes in reactive oxygen species (ROS) and the chlorophyll system. Moreover, Cd-response genes related to stress response, amino acid metabolism, and ROS scavenging were enriched or activated in DK19. Transcriptome and metabolomic analyses highlighted the important role of galactose, lipid (glycerophosphatide metabolism and glycerophosphatide metabolism), and glutathione metabolism in response to Cd stress in buckwheat, which are significantly enriched at the gene and metabolic levels in DK19.

**Discussion:**

The results of the present study provide valuable information for a better understanding of the molecular mechanisms underlying Cd tolerance in buckwheat and useful clues for the genetic improvement of drought tolerance in buckwheat.

## 1 Introduction

### 1.1 Cadmium pollution and influence

Heavy metal pollution in soil is a global problem, which has posed a serious threat to public health. It is reported that in China, the farmland polluted by heavy metals cadmium, lead, arsenic and mercury covers an area of 20 million hm^2^, accounting for about 20% of the total farmland area, of which 14000 hm^2^ of farmland soil is seriously polluted by cadmium (Jiang et al., 2019; Lin et al., 2021). Cd in soil will have adverse effects on crops, agricultural products and groundwater (Qin et al., 2021). Cadmium (Cd) is a non-essential trace heavy metal element for plant growth (Hoseini and Zargari, 2013; Bhattacharya, 2022), it will accumulate in all parts of plants after being absorbed. The toxic effect of Cd on plants is firstly shown as inhibition of root growth, which causes root browning, reduction of lateral root number and root tip death. Secondly, Cd will cause leaf curl, terminal leaf chlorosis and stem growth retardation (Clemens, 2006; Ran et al., 2015; Li et al., 2021a; Meng et al., 2022). At the same time, Cd will replace the essential elements of the active center of enzymes involved in plant biochemical reactions, replace the sulfhydryl groups of proteins and enzymes, change the conformation of macromolecules, cause protein denaturation, and damage the cell membrane, thus affecting the growth and development of plants (Romero-Puertas et al., 2002; Riaz et al., 2021). More dangerous is that Cd accumulated in plants will enter the human body through the food chain, which will cause serious damage to the body (Alengebawy et al., 2021; Rehman et al., 2021).

### 1.2 Detoxification mechanism of plants under cadmium stress

When plants grow in soil rich in Cd, their growth and development are affected comprehensively, and produces a series of tolerance and detoxification mechanisms to control the absorption, transportation and accumulation of Cd to alleviate the toxicity (Zhang et al., 2021b). Plant root epidermis, trichome and cuticle all produce protective tissue to isolate Cd (Feng et al., 2021), and secrete organic acids (citric acid, lactic acid, malic acid) to form a complex with cadmium to inhibit the transmembrane transport of cadmium, and change the pH and EH of the rhizosphere to reduce the migration of soil cadmium into the root (Chen et al., 2019a). Polysaccharides and proteins in the cell wall can bind with Cd to prevent Cd from entering the cell, Cd entering cells will be enriched in vacuoles to reduce the toxicity of cadmium to cell activity (Wei et al., 2021). Chelators such as glutathione, plant chelating peptide and metallothionein in plant cells can bind to Cd to avoid Cd binding to proteins with important physiological functions (Baghayeri et al., 2018).

### 1.3 Application of transcriptomics in the study of cadmium stress in plants

Transcriptome connects gene expression and biological function, and shows a strong advantage in identifying key genes that respond to abiotic stresses during plant growth and development (Tang et al., 2011). Many transcriptome analyses have been conducted to analyze the response of plants to abiotic stress (Dossa et al., 2019; Zhang et al., 2019; Kang et al., 2020), and studies on the response to Cd stress have been carried out in rape, Arabidopsis, maize, rice, and other crops (Rui et al., 2018; Shu et al., 2019). Using transcriptome technology to analyze ceruleuca glauca with different genotypes under Cd stress, found that *NcNramp1* played a key role in the process of plant absorption and accumulation of Cd^2+^ (Milner et al., 2014). The results of transcriptome analysis of winter wheat under cadmium stress deteced 429 up-regulated genes and 998 down-regulated genes, and found that these differential genes were involved in the defense and detoxification mechanism under Cd stress (Xiao et al., 2019). Similarly, sequencing analysis of maize under Cd stress showed that auxin affected the Cd content of maize seedlings, and may improve maize yield and Cd tolerance by regulating auxin signals (Yue et al., 2016).

### 1.4 Application of metabonomics in the study of cadmium stress in plants

Metabolomics is a technology to study the metabolic network of biological systems (Weckwerth, 2003), using metabolomics to explore the response mechanism and metabolic network regulation of plants under abiotic stress is of great significance (Sung et al., 2015; Feng et al., 2020; Nephali et al., 2020; Kumar et al., 2021). After Cd treatment of amaranth, 41 different metabolites were identified, of which 12 differential metabolites were significantly related to three pathways (the synthesis of valine, leucine and isoleucine) (Mei et al., 2014). The metabonomic analysis of Brassica napus by LC-MS proved that the production of reducing agents (monoterpenoids, carotenoids, etc.) plays an important role in promoting the detoxification mechanism and stabilizing the membrane (Mwamba et al., 2020; Wang et al., 2021). In addition, the metabonomic analysis of indica rice grains under Cd stress was carried out by mass spectrometry metabonomic, the results showed that the metabolism of carbohydrates, organic acids and amino acids was affected by Cd stress, and the cysteine content increased, which could improve the tolerance to Cd toxicity (Zeng et al., 2021). Yang et al. (2019) performed a metabolomic analysis of alfalfa under Cd stress to determine the response of proline and free amino acid content to improve its resistance to Cd toxicity.

### 1.5 Response of buckwheat to cadmium stress

Buckwheat, as an important minor cereal, has important nutritional value. However, as a major crop in many remote areas, it has a potential risk of Cd exceeding the standard (Zhang et al., 2017). Therefore, it is of great significance to study the molecular mechanism of buckwheat response to Cd stress. The research on buckwheat under Cd stress mainly focused on physiological characteristics such as seedling growth, subcellular distribution and the transfer coefficient to plants (Mitrus and Horbowicz, 2020; Li et al., 2021b). There are few reports on the use of multiomics integration analyses to study the response law of buckwheat to Cd stress in a deep level, or even to find the core regulatory network sunder it. Therefore, this study uses transcriptome sequencing technology and non-targeted metabolomics technology to integrate and analyze the response mechanism of buckwheat to Cd stress, reveal the key metabolic pathways under it, and provide a new idea for a comprehensive understanding of buckwheat Cd stress response mechanism.

## 2 Materials and methods

### 2.1 Plant material

The samples used in this study were obtained from College of Biological Sciences and Technology, Taiyuan Normal University (Taiyuan, Shanxi, China). The cultivated species named Kuqiao 33 (K33) is an excellent new line developed from Qinghai landrace black Tartary Buckwheat by N ion beam implantation. The field performance is good, the maturity period is consistent, and the lodging resistance and seed falling resistance are good (Zhang et al., 2017).

The novel buckwheat named duoku19 (DK19) is a new perennial species (*F. tatari-cymosum* Q.F. Chen) by crossing annual auto-tetraploid tartary buckwheat and perennial tetraploid *F. cymosum*, using the main agronomic traits (plant height, yield, grain color, etc.) and key molecular markers as screening criteria (Sytar et al., 2014). In a traditional agricultural production model, the establishment of annual crops has both economic and agronomic implications such as high seed and nutrient inputs, ploughing, and may involve a number of sowings each year (Komori et al., 2007).

### 2.2 Growth conditions and cadmium treatment

The experiment was carried out in the greenhouse of Taiyuan Normal University with planting method. Chose buckwheat seeds of similar size sterilized them with 3% NaClO for 10 minutes, then washed them with distilled water for 3 times, and put the seeds on the wet filter paper in the germination box for germination. The culture conditions were: illumination at 25°C for 16 hours, darkness at 25°C for 8 hours, humidity 80%, and 400 μmolm^-2^ s^-1^ intense luminosity. The seedlings were transplanted into a polyethylene pot (contain Hoagland nutrient solution) at the cotyledons and radicle reach 3-5 cm, 12 holes in each pot and 1 plant in each hole for each cultivated variety in the greenhouse. The greenhouse cultivation conditions were 16 hours of light at 25-30°C and 8 hours of dark light at 15-18°C. The air pump supplemented the nutrient solution with oxygen to provide the oxygen content required for root respiration, and the nutrient solution was replaced every 3 days to ensure the consistency of nutrient content in all treatments and before and after each treatment (Zhang et al., 2021a).

Buckwheat with consistent growth was selected and its roots were placed in deionized water for 12 h to remove excess nutrient ions on the root surface. After that, the buckwheat root system was rinsed with deionized water for times, gently wiped dry and immersed in Cd solution (Cd solution, 1 mg/kg) for 2 h under magnetic stirring. Each material was treated with 200 plants. After the adsorption was completed, the buckwheat roots were taken out from the Cd solution, washed with deionized water, and the excess water on the root surface was gently wiped with filter paper. In this study, DK19 and K33 were divided into 6 groups for analysis. The untreated materials were used as the control group (DK19-0 and K33-0), DK19-1 and K33-1 were treated for 7 days, and the materials treated for 14 days were named K19-2 and K33-2.

### 2.3 Determination of chlorophyll content and antioxidant enzyme activity

Phenotype of leaves and chlorophyll content were measured on the 7th and 14th days after treatment, and fresh leaf samples were taken for transcriptome analysis and metabolome analysis. Ten samples were collected at different stages after Cd treatment, and the un-treated samples as the control, the samples were quickly stored in liquid nitrogen. The buckwheat leaf cells were resuspended with 80% acetone solution, incubated for 48 hours, and vibrated at 25 ° at 80 rpm speed in dark culture. Measure the absorbance of the supernatant at 652 nm to calculate the chlorophyll content (Pérez-Patricio et al., 2018).

Take 0.5 g of fresh leaves, put the samples (three replicates) stored at - 80°C into liquid nitrogen precooled mortar, and grind them to powder. Put into a 10 ml centrifuge tube. Add 8 ml 0.05 molL^-1^ sodium phosphate buffer (pH = 7.8) precooled in advance, centrifuge at 10000 rpm for 20 min, and store the supernatant at 4°C for enzyme activity determination (He et al., 2013). The content of malondialdehyde (MDA) was determined by thiobarbituric acid TBA. Peroxidase (POD) activity was determined by guaiacol method (Peter-Katalinić, 2005). The activity of catalase (CAT) was determined by TBA-TCA method (Stewart and Bewley, 1980). Each physiological and biochemical index of each treatment was measured three times, and the results were expressed as the average value.

### 2.4 Untargeted metabolomics of buckwheat leaf tissues

Untargeted metabolomics, a useful approach for the simultaneous analysis of samples with significant phenotypic changes, has been used to detect metabolites after Cd treatment (Gevi et al., 2019). The samples with significant phenotypic changes were used to detect the metabolites after drought treatment. Three experimental replicates were carried out in each treatment stage, and ten materials with consistent growth were used for each replicate. Samples of each group were fully crushed in liquid nitrogen, extracted with 10g leaf samples extract with countryman filter paper. After lyophilization for 7 days, the metabolite samples were ground to powder in a ball mill (MM400, Retsch, Germany) at 30 Hz for 45 s. Then, 0.05–0.1 g powdered sample was extracted as previously description (Ramalingam et al., 2021).

Non-targeted metabolic profiling analyses were profiled by Perkin Elmer 680 GC (Perkin Elmer Inc, Akron, OH, USA) and Q Exactive Focus Orbitrap LC-MS/MS (Thermo Scientific, Waltham, MA, USA). Scanning mass ranged from m/z 100–1000 with an accumulation time of 0.10s. The scanning mode was full MS/ddMS2. The recorded data were processed with compound discoverer (CD) 3.1 software to obtain the mass to charge ratio, retention time, MS/MS2 information of all detected substances. Then, the detected signals were automatically matched through the internally established reference libraries of chemical standard entries of software to predict and identify the metabolite information. The multiple reaction monitoring (MRM) mode with QTRAP 6500+ LC-MS/MS (Shimadzu, Kyoto, Japan) was used for targeted metabolome analyses. The detection window was set to 80 s, and the targeted scanning time was 1.5 s. The original data were processed by Multi Quant 3.0.3 software. The chromatographic column was C18 column (Shim-pack GLSS C18, 1.9UM, 2.1*100, Shimadzu). Mobile phase A and B was 0.04% acetic acid–water solution, and mobile phase B was 0.04% acetic acid–methanol solution. The qualitative and quantitative chromatographic conditions were consistent.

### 2.5 Bioinformatics analysis of metabolome data

Normalize the total peak area by dividing each metabolite in the sample by the total peak area of the sample, so as to normalize the data before analysis. Principal component analysis (PCA), correlation analysis between samples, Orthogonal Partial Least Squares-Discriminant Analysis (OPLS-DA) are carried out by using R software package ropls to classify and discriminate between samples (http://bioconductor.org/packages/release/bioc/html/ropls.htm) (Thevenot, 2016). We combined the multivariate statistical analysis of the VIP value of OPLS-DA and the univariate statistical analysis of the T-test P-value to screen differentially accumulated metabolites (DAMs) among different comparison groups (Wu et al., 2022). Screening criteria FC>1.50, P-value<0.05 and VIP>1 were considered to determine the significant differences between samples. The Kyoto Encyclopedia of Genes and Genomes (KEGG) enrichment analysis was performed on the dam using KOBAS software (Mao et al., 2005).

### 2.6 High throughput transcriptome analysis

After treatment, ten samples were taken for transcriptome analysis with untreated materials as control. Total RNA from plant tissues was extracted using the total RNA extraction kit (Sangon, Shanghai, China, SK1321) and remove genomic DNA with RNase-free DNase-I treated. RNA integrity was evaluated using the Agilent 2100 Bioanalyzer (Agilent Technologies, Santa Clara, CA, USA) (Du et al., 2019). The libraries were constructed using TruSeq Stranded mRNA LTSample Prep Kit (Illumina, San Diego, CA, USA) according to the manufacturer’s instructions and sequenced using Illumina Hi-Seq platform (Bolger et al., 2014). The fluorescent image processing, base-calling, and calculation of quality value were performed by Illumina data processing pipeline 1.4 (Illumina^®^, San Diego, California, USA).

The raw data was filtered and trimmed for low-quality score reads, adaptor, and primer sequences were removed and rawdata (rawreads) were processed using Trimmomatic. The reads containing ploy-N and the low quality reads were removed to obtain the clean reads (Bolger et al., 2014). HISAT2 V2.1.0 was selected to hierarchical indexing for spliced alignment of transcripts to compare transcriptome sequencing reads to the reference genome (Kim et al., 2015). Transcripts Per Million mapped reads (TPM) value of each gene was calculated using cuff links, and there adcounts of each gene were obtained by htseq-count (Trapnell et al., 2010; Anders et al., 2015). DEGs were identified using the DESeq package function estimate Size Factors and nbinomTest. P value < 0.05 and fold Change > 2 or fold Change < 0.5 was set as the threshold for significantly differential expression (Anders and Huber, 2012). Gene Ontology (GO) enrichment and Kyoto Encyclopedia of Genes and Genomes (KEGG) pathway enrichment analysis of DEGs were respectively performed using R based on the hypergeometric distribution using data from http://www.geneontology.org/ and http://www.genome.jp/kegg/, respectively.

### 2.7 Statistical analysis

The statistical analysis was performed using Graph Pad Prism 9 (https://www.graphpad.com/). The experiments were performed with three biological replicates, and plant materials from ten seedlings were pooled for each biological replicate. The statistical significance was determined through a Two-way ANOVA and Tukey’s test. The difference was considered to be statistically significant as **** P<0.0001, *** P ≤ 0.001, ** P ≤ 0.01, and * P ≤ 0.05.

## 3 Results

### 3.1 Physiological analysis of the response of buckwheat seedling to cadmium stress

**Figure 1:**
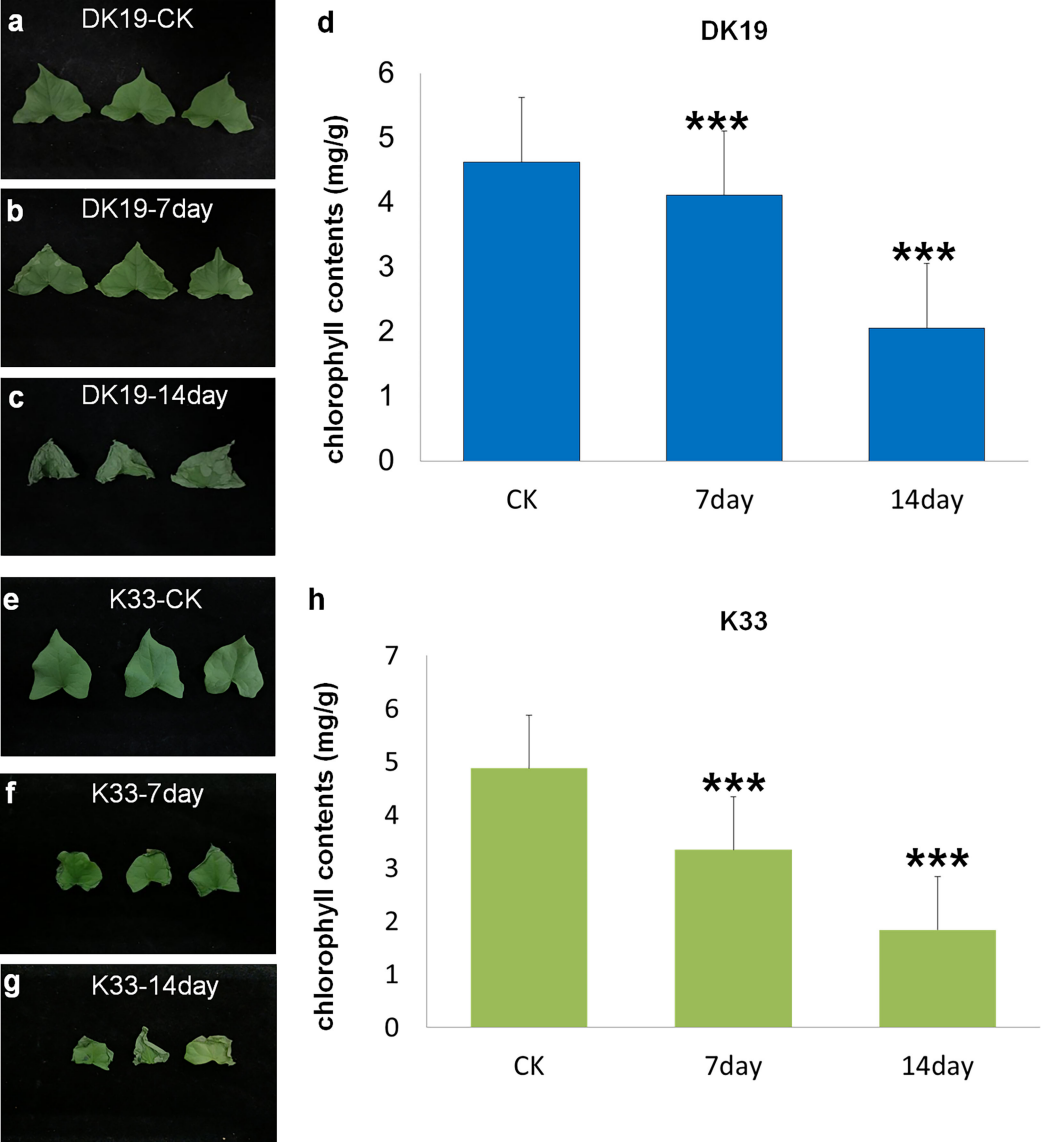
Phenotypic detected before and after Cd stress in DK19 and K33. **(A)** The phenotype of fresh buckwheat leaves was before treatment (CK) in buckwheat DK19. The leaves contain sufficient water, showing good spreading and extension, without wilting and drying. **(B)** The phenotype of fresh buckwheat leaves after seven days of Cd treatment in DK19. Leaves did not show significant variation relative to the control group. **(C)** The phenotype of fresh buckwheat leaves after 14 days of Cd treatment in DK19. The leaves wilted, showing a significant water loss state, but the leaves did not appear withered and yellow. **(D)**, The diversity in chlorophyll contents. The abscissa represents the samples with different treatments, and the ordinate is the chlorophyll content detected, in mg/g. **(E)**, The phenotype of fresh buckwheat leaves was before treatment (CK) in buckwheat K33. The leaves contain sufficient water, showing good spreading and extension, without wilting and drying. **(F)**, The phenotype of fresh buckwheat leaves after seven days of Cd treatment in K33. The leaves wilted, showing a significant water loss state, but the leaves did not appear withered and yellow. **(G)**, The phenotype of fresh buckwheat leaves after 14 days of Cd treatment in K33. The leaves showed significant wilting and serious water loss. A large number of withered and yellow states appeared at the edge of the leaves, and gradually extended to the center. **(H)**, The diversity in chlorophyll contents. The abscissa represents the samples with different treatments, and the ordinate is the chlorophyll content detected, in mg/g. *** P ≤ 0.001.

Buckwheat varieties DK19 and K33 were treated with Cd at the two-leaves seedling stage, and the samples were taken at an interval of one week, the diversity of physiological and phenotypic responses under Cd stress was studied. Compared with normal planting condition (CK), the leaves of DK19 began to curl and wilt from the edge on the 7th day of Cd treatment (**Figure 1B**). On the 14th day, the leaves still maintained a relatively complete shape, and only chlorosis occurred at the edge (**Figure 1C**). In order to further study the effect of Cd stress on the photosynthetic capacity of tartary buckwheat, we detected the chlorophyll content of material DK19 at different Cd treatment times. Results in **Figure 1D** show the variation trend with the extension of Cd stress treatment time. After 7 days of treatment, the chlorophyll content in DK19 decreased, which was 4.112 mg/g. When treated for 14 days, the chlorophyll content in leaves decreased significantly, which was 2.058 mg/g compared with the control.

In K33, there was no significant difference in the leaf phenotype, relative to DK19, in the normal planting condition (**Figure 1E**). All leaves produced significant curly wilting on the seventh day of treatment, which could not be flattened (**Figure 1F**). By the 14th day, the edges of leaves significantly turned yellow and withered, and extended to the center (**Figure 1G**). Significant differences in leaf phenotypic changes were observed between the two materials. It can be seen from the figures that there were no significant difference in chlorophyll content between DK19 (4.623 mg/g) and K33 (4.879 mg/g) leaves under the same conditions without Cd treatment (P<0.05), which was consistent with the leaf phenotype. After seven days of treatment, the chlorophyll content in K33 decreased, which was 3.343 mg/g. The chlorophyll content in K33 decreased significantly. When treated for 14 days, the chlorophyll content in K33 leaves decreased significantly, which was 1.836 mg/g respectively compared with the control.

### 3.2 Difference of malondialdehyde content and antioxidant enzyme system

**Figure 2:**
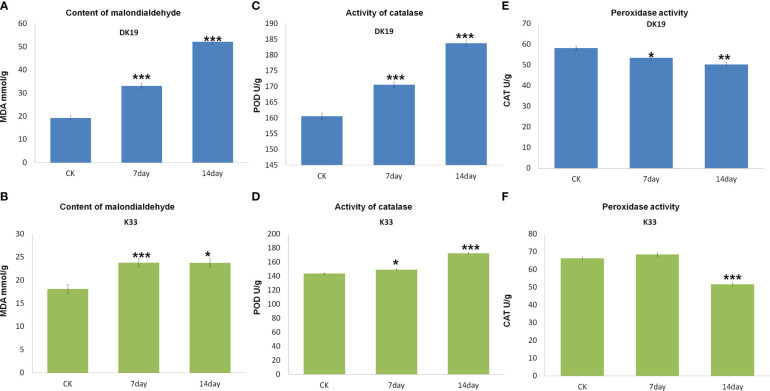
Difference of malondialdehyde content and antioxidant enzyme system between buckwheat DK19 and K33 under Cd stress. **(A)** Difference of MDA content in buckwheat leaves under Cd stress in DK19. **(B)** Difference of MDA content in buckwheat leaves under Cd stress in K33. **(C)** Difference of POD content in buckwheat leaves under Cd stress in DK19. **(D)** Difference of POD content in buckwheat leaves under Cd stress in K33. **(E)** Difference of CAT content in buckwheat leaves under Cd stress in DK19. **(F)** Difference of CAT content in buckwheat leaves under Cd stress in K33. * in green indicated that the difference of the same variety under different treatments is significant (P<0.05). * in black indicated significant difference (P<0.05) between different varieties under the same treatment. ** P ≤ 0.01, *** P ≤ 0.001.

MDA content of two buckwheat varieties increased with the prolongation of cadmium stress treatment time. The MDA content of the two buckwheat varieties increased with increasing Cd stress treatment time. On the 7th and 14th days of treatment, the MDA content in DK19 increased significantly (P < 0.01). The promotion rate on the 7th day was 23.752mmol/g, and the promotion rate on the 14th day was 23.729mmol/g (**Figure 2A**). There was no significant difference in MDA content between K33 and DK19 when untreated (P>0.05). The content was 33.125mmol/g (K33) and 52.168mmol/g (**Figure 2B**) after 7 and 14 days of treatment, respectively. After 14 days of treatment, the MDA content of the two varieties was significantly different from that of the control group (P < 0.001). In different Cd treatment periods, the MDA content in K33 leaves was always higher than that in DK19, indicating that the membrane damage in K33 was more serious than that in DK19. It can be seen from **Figure 2C** that the activity of POD kept up-regulated in the two treatment stages. With the passage of time (**Figure 2B**), the activity of POD in DK19 leaves was significantly up-regulated (P < 0.01) and extremely significantly up-regulated (P < 0.001), while the activity of POD in K33 leaves was significantly lower than DK19 in each period (P < 0.001) (**Figure 2D**). **Figure 2E** and **Figure 2F** show the change of CAT activity in leaves. CAT activity in DK19 leaves was lower than K33 in each period of treatment, and showed significant difference and extremely significant down-regulation in control and treatment for 7 days respectively. CAT activity in K33 leaves was slightly higher than DK19 in treatment for 14 days. It indicated that more drastic changes in CAT activity occurred in DK19 leaves with the passage of treatment time. The changes of MDA content and antioxidant enzyme activity in buckwheat leaf tissue under Cd stress showed that the treatment of Cd stress significantly changed the redox reaction in buckwheat, and there was a significant difference between the two materials.

### 3.3 Statistical description of metabolic analysis of untargeted metabolomics sequencing

**Figure 3:**
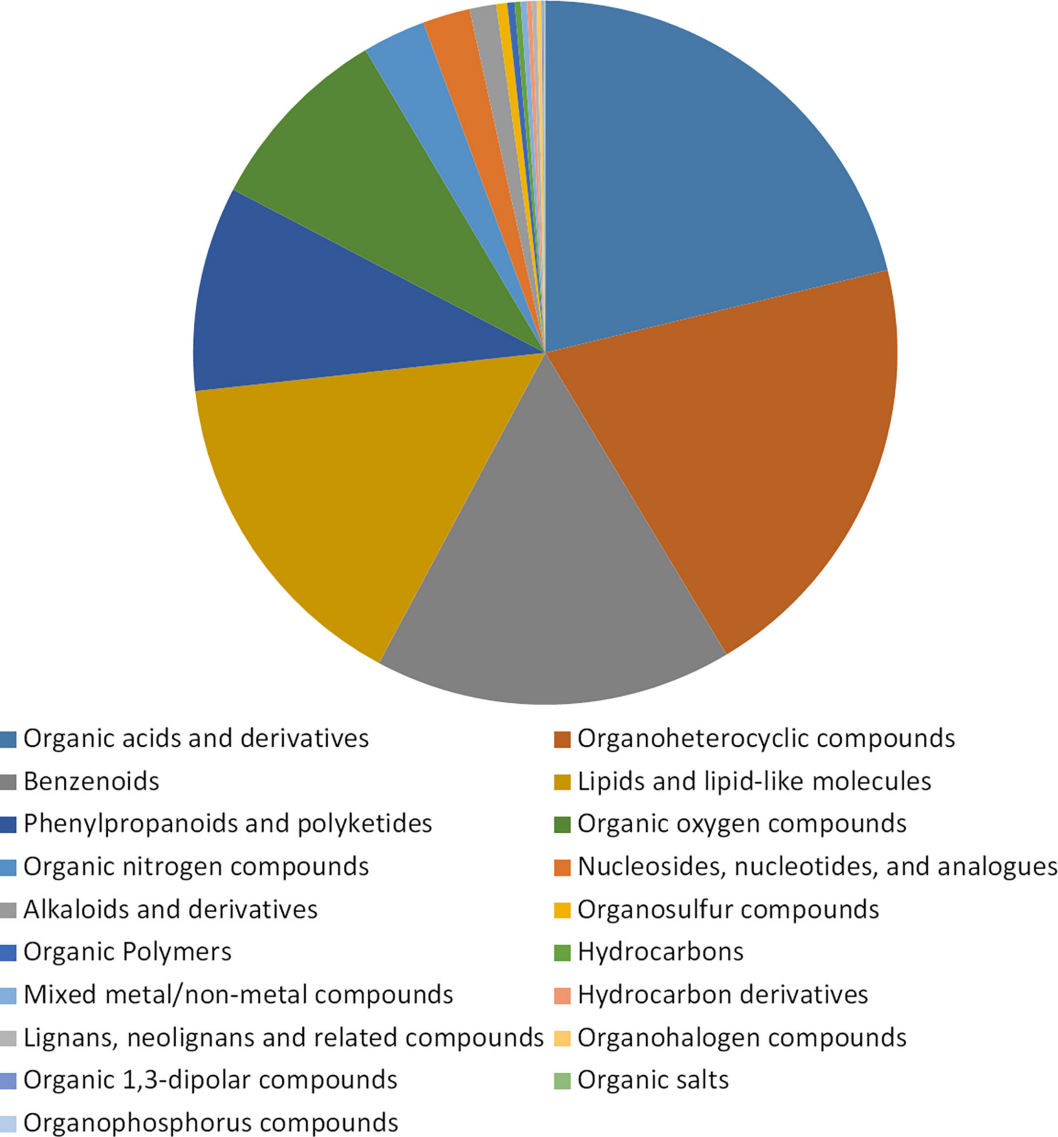
Statistical description of metabolic analysis of buckwheat on Cd response. Classification of the 1798 known identified metabolites in non-targeted metabolome analysis. The ratio of the detected metabolites to the total metabolites in the pie chart. Blocks with different colors indicate different supertypes of metabolite values, and different types are arranged in alphabetical order of names.

Untargeted metabolomics was used to detect the accumulation of metabolites in buckwheat leaves tissues at the period with significant phenotypic variation (day 7 and day 14) after Cd treatment. The content of metabolites in buckwheat leaves without Cd treatment was used as a control in DK19 and K33, respectively. A total of 1798 metabolites were identified in this study, which were divided into 19 categories (**Figure 3**), and the metabolites were annotated according to their chemical structure. Among them, 381 metabolites are organic acids and derivatives. There were 362, 295, 277, 169 and 158 metabolites detected in the categories of organic heterocycles, benzene, lipids and lipid molecules, phenylpropane, polyketones and organic oxygen compounds. The third kind of super categories, like organic nitrogen compounds, nucleosides, nucleotides, and analogues and alkaloids and derivatives, containing 52, 39 and 22 metabolites were detected respectively. In addition, a large number of organo sulfur compounds (9), organic polymers (6), mixed metal/non-metal compounds (5), hydrocarbons (5), hydrocarbon derivatives (4), lignans, neolignans and related compounds (4), organohalogen compounds (4), organic 1, 3-dipolar compounds (1), organic salts (1), organophosphorus compounds (1) were deteced.

A total of 153 classes were identified through an extensive comparison of the literature. Among these classes, the groups with the highest number of metabolites were carboxylic acids and derivatives (324), benzene and substituted derivatives (194), organooxygen compounds (153), and fatty acids (116). In addition, classes containing more than 20 kinds of metabolites include flavonoids (81), steroids and steroid derivatives (67), prenol lipids (59), organonitrogen compounds (52), indoles and derivatives (45), pyridines and derivatives (28), imidazopyrimidines (27), phenol ethers (26), coumarins and derivatives (25), naphthalenes (25), peptidomimetics (25), phenols (22), benzopyrans (21), azoles (20) and glycerophospholipids (20). Rich metabolite types were first detected in buckwheat after cadmium stress treatment in this study. Among 153 types, 27 types detected extensive variation before and after treatment. Among them, amino acid metabolism, antioxidant metabolism and energy metabolism all contain a large number of variations, which will be analyzed later in this study.

### 3.4 Screen of differentially accumulated metabolites

**Figure 4:**
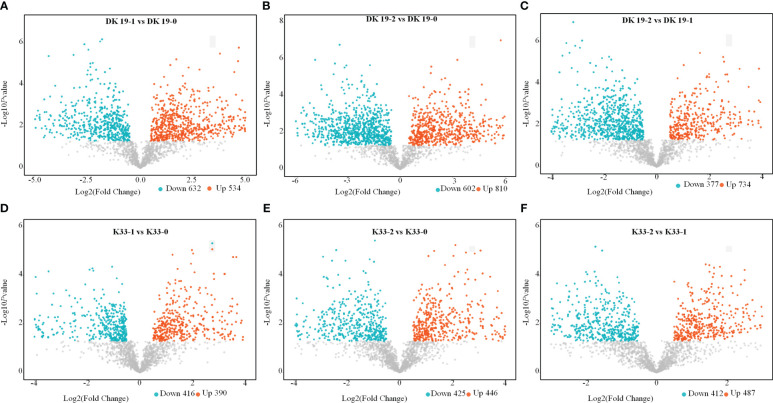
Screen of differentially accumulated metabolites (DAMs), generated using untargeted metabolomics sequencing. The volcano plot presents the expression of the DAMs in different treatments, the horizontal axis is Log2 (Fold Change) value, and the vertical axis is - Log10*p*value, the red dots represent up-regulated metabolites, and the blue dots represent down-regulated metabolites. **(A–C)** DAMs in DK19 between different stages of treatment. **(D–F)** DAMs in K33 between different stages of treatment.

Through the screening of metabolites, differentially accumulated metabolites (DAMs) with criteria FC>1.50, P-value<0.05 and VIP>1 were considered to determine the significant differences between samples (Punia et al., 2021). In DK19, compared with CK, 534 metabolites were up-regulated and 632 metabolites were down-regulated after 7 days of treatment (**Figure 4A**). In the second treatment period (14 days), 810 metabolites were up-regulated and 602 metabolites were down-regulated, taking the materials before treatment as the control (**Figure 4B**) and 734 up-regulated metabolites and 377 down-regulated metabolites were identified between the two stages of the Cd treatment (**Figure 4C**). Correspondingly, in K33, 390 up-regulated metabolites and 416 down regulated metabolites were detected in the first stage of treatment (7 days) relative to that before treatment (**Figure 4D**); On the fourteenth day of treatment (the second stage), 448 up-regulated metabolites and 425 depressed metabolites were detected relative to the control group (**Figure 4E**); Between the two treatments, the up-regulated and down-regulated metabolites were 487 and 412, respectively (**Figure 4F**). Corresponding to the observed phenotypic variation, with the dramatic change of phenotype, the metabolism in buckwheat cells changed dramatically under Cd stress. There were significant differences in the trends of these differential metabolites between the two materials, shows that DK19 has undergone drastic metabolic changes to respond to Cd stress, thus improving the resistance.

**Figure 5:**
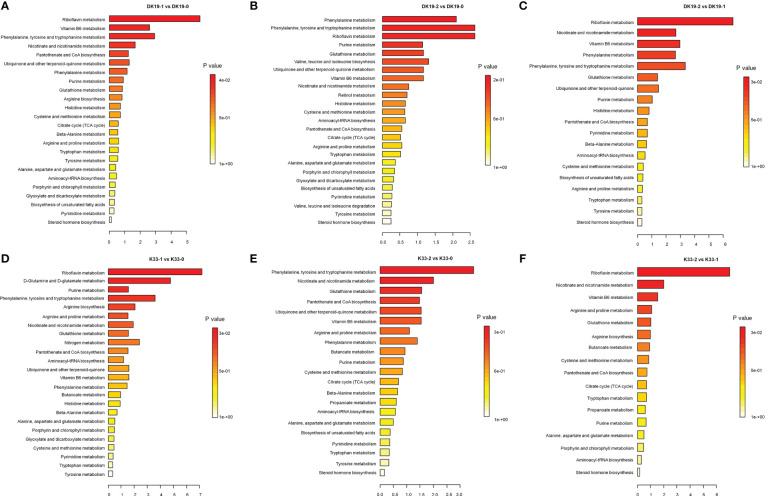
Screen of differentially accumulated metabolites (DAMs), generated using untargeted metabolomics sequencing. The Top KEGG pathways of all DAMs between different stages of treatment. The abscissa is the percent of differentially accumulated metabolites in different KEGG, and the ordinate is the type of KEGG enrichment. The darker the color, the higher the *p* value. **(A–C)** DAMs in DK19 between different stages of treatment. **(D–F)** DAMs in K33 between different stages of treatment.

The Kyoto Encyclopedia of Genes and Genomes (KEGG) enrichment analysis was performed on the DAMs using KOBAS software. KEGG enrichment was carried out for DAMs between different treatments, and the 25 top pathways are shown in **Figure 5**. In DK19, in the first stage of treatment, the pathways which enrich the most metabolites are Riboflavin metabolism, Vitamin B6 metabolism, Phenylalanine, tyrosine and tryptophan, Nicotinate and nicotinamide metabolism (**Figure 5A**). In the second treatment period (14 days), the top five KEGG pathways, Phenylalanine metabolism, Phenylalanine, tyrosine and tryptophan, Riboflavin metabolism, Purine metabolism and Glutathione metabolism are enriched, taking the materials before treatment as the control (**Figure 5B**). Between the two stages after Cd treatment, DAMs were enriched in Riboflavin metabolism, Nicotinate and nicotinamide metabolism, Vitamin B6 metabolism, Phenylalanine metabolism and Phenylalanine, tyrosine and tryptophan (**Figure 5C**). For material K33, in the first stage of treatment, the pathways enriched to the most metabolites were Riboflavin metabolism, D-Glutamine and D-glutamate metabolism, Purine metabolism, Phenylalanine, tyrosine and tryptophan and Arginine biosynthesis (**Figure 5D**). In the second stage, there were differences pathways between the DK19 and K33, except for a enriched pathway Phenylalanine, tyrosine and tryptophan were enriched in two materials, four pathways, Nicotinate and nicotinamide metabolism, Glutathione metabolism, Pantothenate and CoA biosynthesis were special enriched in K33 (**Figure 5E**). Between the two stages of treatment, the metabolites were similarly enriched in three pathways (Riboflavin metabolism, Nicotinate and nicotinamide metabolism and Vitamin B6 metabolism), between the two materials (**Figure 5F**).

The metabolite changes of the two materials after Cd treatment showed consistency. For example, among the pathways with the most enriched genes, the three pathways Riboflavin metabolism, Nicotinate and nicotinamide metabolism and Phenylalanine, tyrosine and tryptophan were significant present almost every period. In addition, among other significantly enriched pathways, many pathways were significantly enriched in the two materials at different periods. This result showed that different buckwheat materials, whether resistant or sensitive, showed similar metabolic changes after Cd treatment, which may be the basis of buckwheat tolerance. On the other hand, the difference of metabolite enrichment pathways between the two materials in the same period also indicates the basis of the resistance difference between the two materials. It is suggested that the differences of specific metabolic pathways may be the basis of resistance differences among materials on the basis of the consistent overall trend.

**Figure 6:**
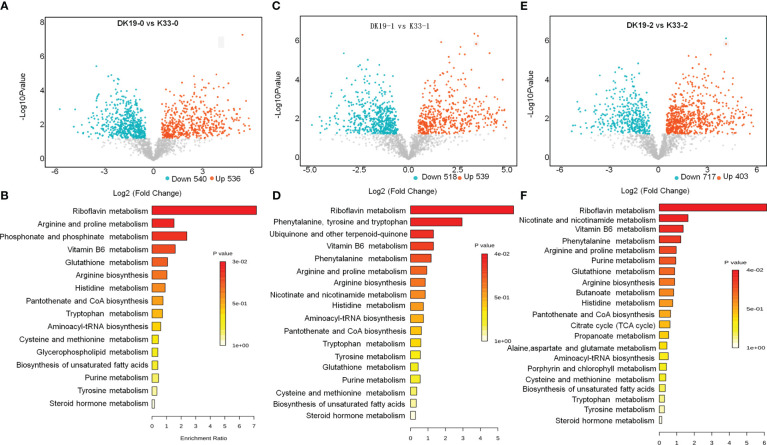
Screen of differentially accumulated metabolites (DAMs) at the same stages between DK19 and K33, generated using untargeted metabolomics sequencing. **(A, C, E)** Identification of differentially accumulated metabolites (DAMs) between DK19 and K33 at the control, 7days after the treatment and 17days after the treatment, generated using untargeted metabolomics sequencing technology. The volcano plot presents the expression of the DAMs in different treatments, the red dots represent up-regulated metabolites, and the blue dots represent down-regulated metabolites. **(B, D, F)** KEGG pathways of all DAMs between the DK19 and K33 at the same stages. The abscissa is the percent of differentially accumulated metabolites in different KEGG, and the ordinate is the type of KEGG enrichment. The darker the color, the higher the *p* value.

Subsequently, we analyzed the differential metabolites of two materials (DK19 and K33) at the same treatment stage (**Figure 6**). As shown in **Figure 6A**, there were 536 metabolites up-regulated and 540 metabolites down-regulated in DK19 relative to K33. These metabolites were mainly enriched in 16 pathways including riboflavin metabolism, arginine and proline metabolism, phosphonate and phosphinate metabolism, vitamin B6 metabolism and glutathione metabolism (**Figure 6B**). In the first period (7 days) of treatment, 539 metabolites were up-regulated and 518 metabolites were down-regulated in DK19, compared with K33 (**Figure 6C**). These metabolites were mainly enriched in 18 pathways, of which riboflavin metabolism, phenylalanine, tyrosine and tryptophan, ubiquinone and other terpenoid quinone, vitamin B6 metabolism and phenytalanine metabolism were significantly enriched (**Figure 6D**). After 14 days of treatment, 403 up-regulated metabolites and 717 down-regulated metabolites were detected in DK19, and K33 was used as control (**Figure 6E**). KEGG enrichment showed that these differentially expressed metabolites were significantly enriched in riboflavin metabolism and 20 pathways such as nicotinate and nicotinamide metabolism, vitamin B6 metabolism, phenytalanine metabolism and arginine and proline metabolism (**Figure 6F**). These results showed that there were some metabolic differences between the two materials before Cd treatment, which may be the basis of the difference in resistance between the two materials. In a period defined by treatment, the two materials had significant differences in metabolite accumulating in arginine and proline metabolism, phosphonate and phosphinate metabolism, phenytalanine, tyrosine and tryptophan, ubiquinone and other terpenoid-quinone, glutathione metabolism, phenytalanine metabolism and nicotinate and nicotinamide metabolism pathways on the basis of original differences. In the second period, the differences to be negotiated are nicotinate and nicotinamide metabolism, phenytalanine metabolism and glutathione metabolism, which continue the change trend of the first period, and purine metabolism, butanoate metabolism, citrate cycle (TCA cycle) and alaine, aspartate and glutamate metabolism, which change significantly in this period. The comparison of the two materials at the same treatment stage showed that at the metabolic level, the resistance to Cd stress had different changes, and the phenotypic differences were obtained.

### 3.5 Identification and functional analysis of differentially expressed genes

**Figure 7:**
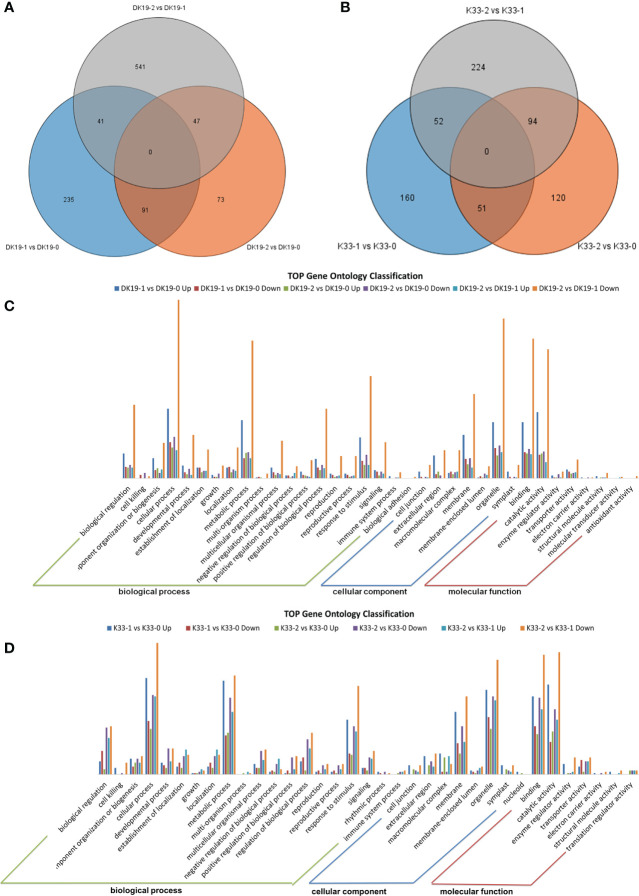
The quality and quantity of the RNA sequencing, generated using high-throughput deep sequencing technology; **(A)** Venn diagrams of differentially expressed genes (DEGs) among the three stages. DK19 and K33 represent two materials, DK9-0 and K33-0 are the control without Cd treatment, DK19-1 and K33-1 are the test results after 7 days of treatment, and DK19-2 and K33-2 are the test results after 14 days of treatment. The selected gene is average TPM> 0.5. **(B)** Relationship between samples in three biological replicates and summary of expressed genes in different treatments. **(C, D)** Venn diagrams of differentially expressed genes (DEGs) among the four stages. 0D represents the control without IAA treatment, 1D represents the sample after 1 day of treatment, 2D represents the sample after 2 days of treatment, and 3D represents the sample after 3 days of treatment. The selected gene is average TPM> 0.5.

High throughput transcriptome analysis was performed using the same materials, a total of 122.58 GB of clean data was obtained, the transcript abundance of each gene was evaluated by Transcripts Per Million mapped reads (TPM) on the basis of the number of uniquely mapped reads that overlapped with exon regions. A total of 29,739 expressed genes were identified. The genes which were detected twice in three replicates and with an average expression of more than 0.5 in TPM were selected as expression gene, and the differentially expressed genes at different stages of treatment were detected according to the standard of P value < 0.05 and fold Change > 2 or fold Change < 0.5. A Venn diagram was created to identify the expressed genes of each material, 367 (DK19-1 *vs.* DK19-0), 211 (DK19-2 *vs.* DK19-0), 629 (DK19-2 *vs.* DK19-1), 263 (K33-1 *vs.* K33-0), 265 (K33-2 *vs.* K33-0) and 370 (K33-2 *vs.* K33-1) differentially expressed genes (DEGs) were identified, respectively (**Figures 7A, B**). Genes with continuous changes in the three periods were not detected in the two materials, and 91 genes were changed in the two periods of treatment, compared with the control, in DK19. The expression of 41 genes changed in the first stage of treatment compared with the control and between the two periods of treatment. 47 genes had expression changes in the second period of treatment, whether compared with the control or the first period. The number of differentially expressed genes distributed in k33 was 51 (K33-1 *vs.* K33-0 compared with K33-1 *vs.* K33-0), 52 (K33-1 *vs.* K33-0 compared with K33-2 *vs.* K33-1) and 94 (K33-2 *vs.* K33-0 compared with K33-2 *vs.* K33-1).

In order to further analyze the DEGs, Gene Ontology (GO) classification analysis of the identified unique DEGs was performed. All the DEGs were divided into the biological process, cellular component, and molecular function categories, the top 35 GOs in DK19 were shown in **Figure 7C**, and top 35 GOs in K33 were shown in **Figure 7D**. In DK19, five GOs, biological regulation, cellular process, metabolic process, response to stimulus, belong to biological process, with the most genes enriched. Three GOs, extracellular region, membrane, organelle, belong to cellular component, with the most genes enriched. Binding and catalytic activity were two GOs with the much genes enriched in molecular function. In most GOs, the combination of DK19-2 *vs.* DK 19-0 down enriched the most differentially expressed genes, followed by DK 19-1 *vs.* DK 19-0 up and DK 19-2 *vs.* DK 19-0 down. Similar GO enrichment trends were also detected in K33, and the GOs with a large number of genes include cellular process, metabolic process, reproductive process, macromolecular complex, membrane-enclosed lumen, binding, catalytic activity. The combinations containing the most genes were K33-2 *vs.* K33-1 Down, K33-1 *vs.* K33-0 up and K33-2 *vs.* K33-0 down. The results of GO analysis showed that the two materials had significant changes in cell composition and expression regulation during cadmium treatment. Especially in the first stage of change, many differentially expressed genes were detected in both materials, which was consistent with the stage of stress response. On the basis of some similar trends, there are also significant differences, even showing opposite expression changes in the two materials, which are related to the changes of phenotypes and physiological indicators.

**Figure 8:**
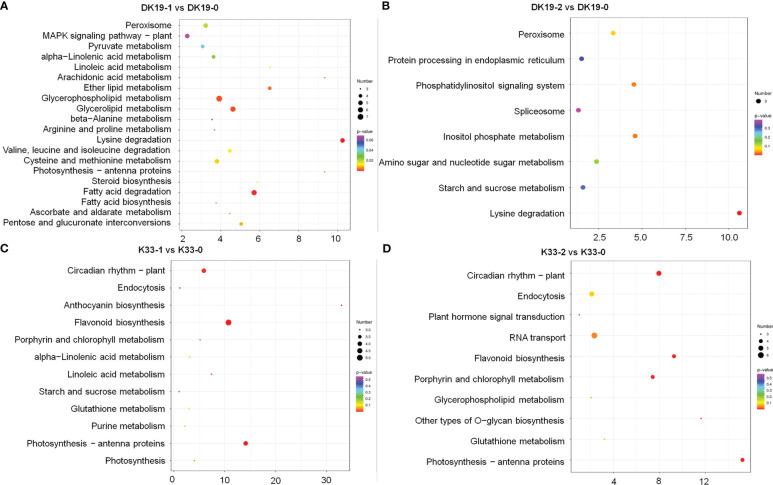
KEGG analysis of differentially expressed genes (DEGs) between DK19 and K33 at the same stages, generated using high-throughput deep sequencing technology. **(A)** The KEGG pathways of all DEGs between the DK19-1 and DK19-0. The abscissa is the percent of differentially accumulated metabolites in different KEGG, and the ordinate is the type of KEGG enrichment. The darker the color, the higher the p value. **(B)** The KEGG pathways of all DEGs between the DK19-2 and DK19-0. The abscissa is the percent of differentially accumulated metabolites in different KEGG, and the ordinate is the type of KEGG enrichment. The darker the color, the higher the p value. **(C)** The KEGG pathways of all DEGs between the K33-1 and K33-0. The abscissa is the percent of differentially accumulated metabolites in different KEGG, and the ordinate is the type of KEGG enrichment. The darker the color, the higher the p value. **(D)** The KEGG pathways of all DEGs between the K33-2 and K33-0. The abscissa is the percent of differentially accumulated metabolites in different KEGG, and the ordinate is the type of KEGG enrichment. The darker the color, the higher the p value.

When subjected to a Kyoto Encyclopedia of Genes and Genomes (KEGG) pathway analysis, using Q-value less than 0.05 as the significant enrichment threshold to all the DEGs for KEGG pathway, the significantly enriched 15 pathways are shown in **Figure 8**. All the pathways were divided into cellular processes, environmental information processing, genetic information processing, metabolism, and organismic systems. In DK19, at the 7^th^ day, the differentially expressed genes were mainly enriched in pathways such as amino acid metabolism (13), carbohydrate metabolism (22), lipid metabolism (16). After two weeks treatment, the DEGs were significantly enriched in carbohydrate metabolism (12) and lipid metabolism (7). Between the two stages of treatment, DEGs were enriched in signal transduction (25), translation (15), amino acid metabolism (14), carbohydrate metabolism (26), lipid metabolism (16), the most DEGs were enriched in carbohydrate metabolism and signal transduction, in particularly. In the lines K33, the most enriched KEGGs were amino acid metabolism (12) and biosynthesis (11) of DEGs between K33-1 *vs.* K33-0, the most enriched KEGG was carbohydrate metabolism (11) between K33-2 *vs.* K33-0, carbohydrate metabolism (16), lipid metabolism (15) and translation (11) were the most three KEGGs between K33-2 *vs.* K33-1. By comprehensively comparing the enrichment of differential expression between the two lines in the three periods, it is obvious that through KEGG analysis, the differentially expressed genes are significantly enriched in carbohydrate metabolism, lipid metabolism, and translation. The correlation analysis between the KEGG enriched by differentially expressed genes and the KEGG enriched by differentially expressed metabolites found that the relevant pathways were mainly in the arginine and proline metabolism, the citrate cycle (TCA cycle) and the metabolism of alanine, aspartic acid and glutamic acid, as well as the metabolism of amino acid, carbohydrate metabolism, lipid metabolism and translation regulation, and were further analyzed in the follow-up study.

### 3.6 Key metabolic pathways identified by joint analysis

According to the previous research results, the obtained differential genes were uploaded to GO database and KEGG database for functional annotation and pathway enrichment. The results showed that the differential genes were significantly enriched in nitrogen metabolism, galactose metabolism, phenylpropanoid biosynthesis, glutathione metabolism, carbohydrate metabolism, linolenic acid metabolism and other metabolic pathways. The metabolomic analysis of buckwheat samples by non-targeted metabolomics showed that the main metabolic pathways for the enrichment of differential metabolites were the biosynthesis of secondary metabolites, amino acid biosynthesis, galactose metabolism, glycerophosphatide metabolism, and so on. Combined with transcriptome and metabolome data, galactose metabolism, lipid metabolism (glycerophosphatide metabolism and glycerophosphatide metabolism) and glutathione metabolism, which are significantly enriched at gene level and metabolic level, are further analyzed.

**Figure 9:**
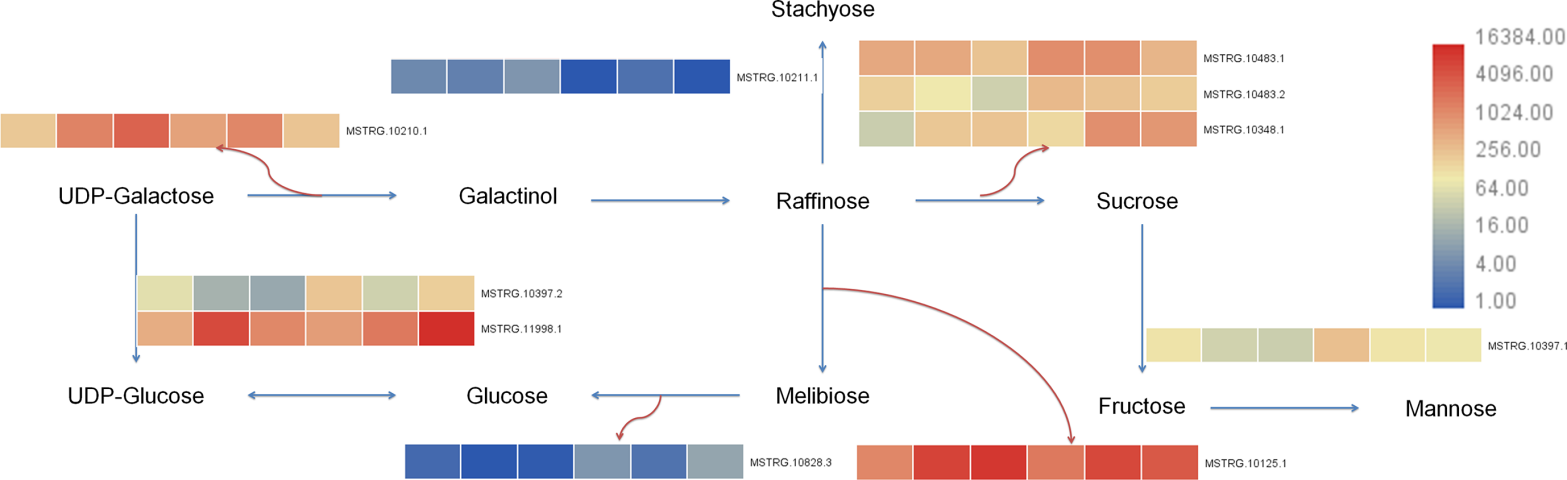
Galactose metabolism pathway in buckwheat leaves under Cd stress. The sample number in the order from left to right in the heat map: DK9-0, DK19-1, DK19-2, K33-0, K33-1 and K33-2.

Transcriptome of this study showed that 25 differential genes were enriched in galactose metabolism (galactose metabolism, ko00052), 7 differential genes were enriched in fructose and mannose metabolism (fructose and mannose metabolism, ko00051), and 43 differential genes were enriched in starch and sucrose metabolism (starch and sucrose metabolism, ko00500) after 7 days of Cd stress. After 14 days of Cd stress, 20 differential genes were enriched in galactose metabolism, 8 differential genes were enriched in fructose and mannose metabolism, and 30 differential genes were enriched in starch and sucrose metabolism. The metabolome results showed that 6 differential metabolites were enriched in galactose metabolism, 3 differential metabolites were enriched in fructose and mannose metabolism, and 1 differential metabolite was enriched in starch and sucrose metabolism. We uploaded the differential genes and metabolites to the KEGG website for enrichment analysis. In this study, the selected 10 candidate genes were tested to verify the galactose metabolic pathway, and the results are shown in **Figure 9**.

**Figure 10:**
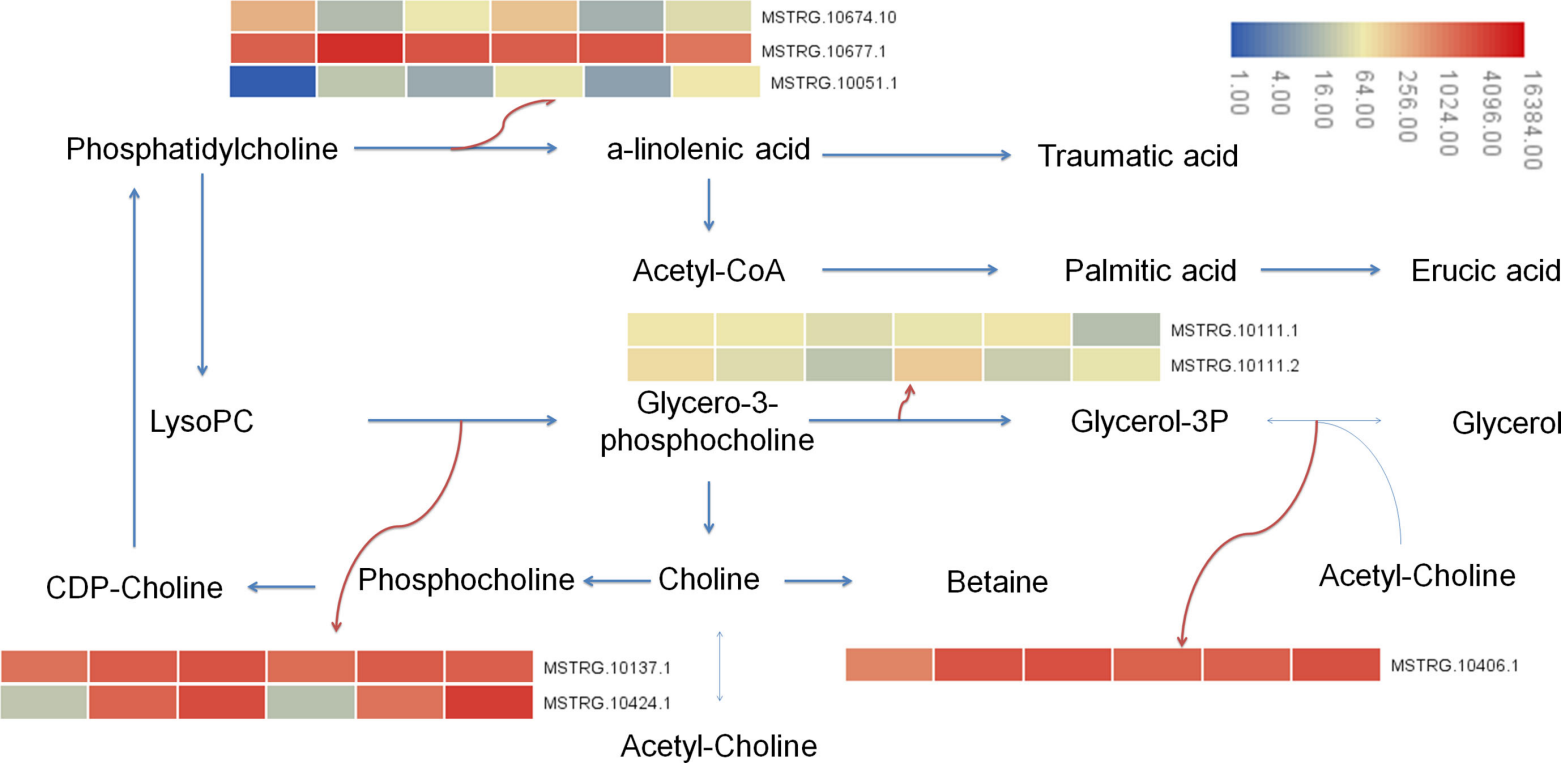
Lipid metabolism pathway in buckwheat leaves under Cd stress. The sample number in the order from left to right in the heat map: DK9-0, DK19-1, DK19-2, K33-0, K33-1 and K33-2.

In this study, the transcriptional results showed that after 7 days of Cd stress, 15 differential genes were enriched in glycolipid metabolism (ko00561), 12 differential genes were enriched in glycerophospholipid metabolism (ko00564), and 20 genes were enriched in α- Linolenic acid metabolism (alpha linolenic acid metabolism, ko00592). A total of 9 differential genes were enriched in glycerol metabolism, 13 differential genes were enriched in glycerol phospholipid metabolism, and 20 genes were enriched in α-Linolenic acid metabolism. Metabolomic results showed that 2 differential metabolites were enriched in glycerophospholipids metabolism, 3 differential metabolites were enriched in glycerophospholipids metabolism, and 1 metabolite was enriched in α-Linolenic acid metabolism. Metabolites involved in lipid metabolism include glycerol triphosphate (1.69 times), lecithin (1.53 times), palmitic acid (0.45 times), betaine (1.28 times), etc. The up regulation of genes was the main reason for the increase of lysophosphatide and glycerol-3-phosphate, and the down regulation of genes were the main reason for the decrease of palmitic acid content (**Figure 10**).

## 4 Discussion

### 4.1 Effects of cadmium stress on plant

Excess Cd in soil has become one of the major environmental problems of global concern, because once Cd is discharged into the environment, it is difficult to collect and treat, and it has super durability (Li et al., 2019; Naeem et al., 2019; Rahman and Singh, 2019). Cd pollution in farmland soil seriously threatens agricultural productivity, food safety and human health (Sarwar et al., 2010; Wang et al., 2019). Therefore, exploring the key genes of low Cd accumulation in buckwheat is helpful to screen buckwheat with low Cd accumulation, so as to solve the important technical guarantee and industrial foundation of buckwheat safety production (Tiryakioglu et al., 2006; Borges et al., 2019). In addition, high-throughput sequencing can analyze the expression regulation under specific abiotic stress, so as to study the response of the body to changes in internal and external environment, and study the changes of gene expression regulation and metabolic regulation network (Chen et al., 2019b; Guo et al., 2020). Combining the advantages of transcriptome and metabolome can solve the deep-seated problems (Szymański et al., 2020).

### 4.2 Advances in molecular mechanisms related to cadmium accumulation and tolerance in plants

The results of this study are also consistent with those of other crops. Under Cd stress, the growth of leaves of seedlings slowed down, and even withered, withered and necrotic after treatment. Under Cd stress for 7 and 14 days, although the growth of DK19 was inhibited to some extent, K33 was obviously more severely poisoned (**Figure 1**). It can be seen that DK19 is a very good material for studying Cd resistance. Based on metabonomics and transcriptomics methods, this anti Cd mechanism of DK19 can be further analyzed. 632 (7 days of treatment) and 602 (14 days of treatment) up-regulated and enriched metabolites and 534 (7 days of treatment) and 810 (14 days of treatment) down-regulated and enriched metabolites were detected in resistant material DK19 during the two periods of significant surface changes, corresponding to relatively few differentially enriched metabolites detected in sensitive material K33 (**Figure 4**). Through GO annotation and KEGG enrichment of differential genes and metabolites, it was found that cadmium stress affected carbon metabolism, nitrogen metabolism, galactose metabolism, plant signal transduction, glutathione metabolism, fatty acid metabolism and phenylpropanoid biosynthesis and other metabolic processes, which may be a stress response of buckwheat under cadmium stress (**Figure 5**). These induce oxidative stress and osmotic stress, and the change trend of carbohydrate content is consistent with the research in rice, wheat and other crops (Liu et al., 2018; Li et al., 2020).These induce oxidative stress and osmotic stress, and the change trend of carbohydrate content is consistent with the research in rice, wheat and other crops (Liu et al., 2018; Li et al., 2020).

Hossain et al. believed that the resistance of plants to a certain type of heavy metals means that plants can normally survive in a specific heavy metal environment with high content, without growth stagnation or even death. SOD, POD, GSH and other factors in plants can repair cell functions and maintain homeostasis by activating the antioxidant system to remove stress induced free radicals (Hossain and Komatsu, 2013). In recent years, many studies have used transcriptome analysis to study Cd responsive genes of different species, and identified a large number of differentially expressed genes (DEGs) responding to Cd stress. These differentially expressed genes are involved in many processes such as cell wall biosynthesis, GSH metabolism, TCA cycle and antioxidant system, which may play a key role in cell wall binding, vacuole isolation and detoxification (Peng et al., 2015; Xu et al., 2015). Similarly, transcriptome analysis showed that many differentially expressed genes in response to Cd stress were involved in cell wall modification, heavy metal transport and phenylacetone biosynthesis, respectively (Feng et al., 2018). At present, there have been many studies on metabolite analysis in plant response to various stresses, including but not limited to nutrient deficiency (Hirai and Saito, 2004), mineral toxicity (Uraguchi et al., 2011), temperature and oxidative stress and osmotic stress (Gong et al., 2005). In addition, mass spectrometry metabonomics was used for the first time to conduct metabonomic analysis of indica rice grains under Cd stress. The results showed that carbohydrate, organic acid and amino acid metabolism were affected by Cd stress, and the content of cysteine increased, which could improve the tolerance to Cd toxicity (Zeng et al., 2021).

### 4.3 Effects of cadmium stress on plant antioxidant system

Cd also has negative effects on physiological and biochemical processes such as photosynthetic efficiency and enzyme activity of plants (Mostofa et al., 2015). The accumulation of Cd in plants leads to the impairment of amino acid biosynthesis, inhibition of enzyme activity, oxidative stress response, interference in mineral nutrition absorption, and metabolic imbalance. Cd can destroy cell membranes, biomolecules, and organelles in plants by increasing the production of reactive oxygen species (ROS). Plant resistance to Cd stress depends on their ability to scavenge ROS. In this study, we detected significant content changes in malondialdehyde (MDA), peroxidase (POD) activity and activity of catalase (CAT) on the 7th and 14th days of cadmium treatment of the two materials. Through the analysis of transcriptome and metabolomic data, enriched differential metabolites and differentially expressed genes detected pathways including galactose metabolism, lipid metabolism (glycerol phospholipid metabolism and glycerol phospholipid metabolism) and glutathione metabolism. Ten key genes with different expression were identified in Galactose metabolism pathway and nine key genes with different expression were identified in Lipid metabolism pathway. The further identified differential metabolites and related core genes are also consistent with previous studies.

Previous studies have shown that oxidative stress under Cd stress enhances the chemical reactivity in plants, and higher levels of reactive oxygen species will lead to protein and nucleic acid degradation, lipid peroxidation, and cell membrane damage (Kaya et al., 2020; Hayat et al., 2021). Scavenging reactive oxygen species is an important defense mechanism for plants to resist external stress. Plants have evolved antioxidant mechanisms and ascorbic acid glutathione mechanisms to avoid the harmful effects of reactive oxygen species. Among them, the main antioxidants are superoxide dismutase (SOD), catalase (CAT) and peroxidase (POD) and ascorbic acid peroxidase (APX), glutathione reductase (GR) and dehydroascorbic acid reductase (DHAR) of ascorbic acid glutathione system (Dat et al., 2000; Sharma et al., 2012; Sachdev et al., 2021).

## 5 Conclusion

Using transcriptome and metabolome data, we identified a large number of candidate genes and metabolites involved in the key biological pathways of buckwheat Cd stress. Our results show that the regulatory mechanism of Buckwheat under Cd stress may not only be a single gene or metabolite, but a complex regulatory and signaling mechanism. How these candidate genes or metabolites participate in buckwheat cadmium stress deserves further study.

## Data availability statement

The datasets presented in this study can be found in online repositories. The names of the repository/repositories and accession number(s) can be found below: NGDC (https://ngdc.cncb.ac.cn/), accession number SRA716568.

## Author contributions

DD conceived and designed the experiments. DH and CW wrote the manuscript. JZ and LQ performed the experiments. YH and JZ analyzed the data. DD and CW revised the manuscript. CW and DD contributed equally to the paper. All authors contributed to the article and approved the submitted version.
